# Density‐dependent diel activity in stream‐dwelling Arctic charr *Salvelinus alpinus*

**DOI:** 10.1002/ece3.2177

**Published:** 2016-05-13

**Authors:** Amy Fingerle, Nicolas Larranaga, Stefán Óli Steingrímsson

**Affiliations:** ^1^ Department of Aquaculture and Fish BiologyHólar University CollegeSauðárkrókurIceland

**Keywords:** Activity rate, resource partitioning, rivers, salmonids, temporal segregation

## Abstract

Intraspecific competition plays a significant role in shaping how animals use and share habitats in space and time. However, the way individuals may modify their diel activity in response to increased competition has received limited attention. We used juvenile (age 1+) Arctic charr *Salvelinus alpinus* to test the prediction that individuals at high population density are more active and distribute their foraging activity over a greater portion of the 24‐h cycle than individuals at low population density. Individually tagged fish were stocked in seminatural stream enclosures at low (2 fish/m^2^) and high (6 fish/m^2^) density. During each of two 2‐week experimental rounds, activity of all fish within each enclosure was recorded every 3 h over seven 24‐h cycles. At high density, fish were more active and distributed their activity over a greater portion of the 24‐h cycle, with increased activity particularly at crepuscular times. Fluctuations in ecological conditions (e.g., water temperature and light intensity) also affected activity. Fish at high density grew as fast as fish at low density. This study demonstrates that individuals exhibit a degree of behavioral flexibility in their response to changes in ecological conditions and suggests that intraspecific competition can cause animals to modify temporal aspects of their activity to gain access to resources and maintain growth.

## Introduction

Animals share and compete for resources in both space and time and frequently adopt strategies that reduce conflict among potential competitors (Schoener 1974; Chesson 2000). Coexistence of ecologically similar species is facilitated by, for example, spatial segregation through habitat selection (Rosenzweig 1987; Kneitel and Chase 2004) and temporal segregation through timing of activity (Kronfeld‐Schor and Dayan 2003). Animals also compete with conspecifics for access to resources such as food (e.g., Milinski 1982; Lewis et al. 2001), shelters (e.g., Davey et al. 2009), and mates (West‐Eberhard 1983; Weir et al. 2011). Spatial responses of animals to intraspecific competition are commonly examined, for example, in the context of territoriality (Hixon 1980; Adams 2001; López‐Sepulcre and Kokko 2005) and population distribution (Fretwell and Lucas 1970; Rodenhouse et al. 1997), but how individuals may modify their diel activity in response to increased competition has received less attention (Guénard et al. 2012).

Diel activity – the allocation of activity and rest within the 24‐h cycle (Reebs 2002; Kronfeld‐Schor and Dayan 2003) – provides insight into how animals exploit and share habitats and resources in time. Diel activity patterns are influenced by a range of ecological factors such as predation risk (Lima and Bednekoff 1999), prey availability (Brown et al. 2001), temperature (Avenant and Nel 1998), and photoperiod (Kolowski et al. 2007). Importantly, animals may also modify their diel activity in response to competition by adjusting their overall rate of activity, the timing of their activity, or both. To date, studies of how interspecific competition may affect activity patterns primarily focus on temporal partitioning of habitats and resources (e.g., Albrecht and Gotelli 2001; Harrington et al. 2009), whereas at the intraspecific level, more emphasis is placed on whether animals modify their overall activity rates in the presence of conspecifics (e.g., Coulombe et al. 2008; Vera et al. 2011; Guénard et al. 2012).

Intraspecific competition is invariably linked to population density; competition increases as more individuals compete for the same resource (Amundsen et al. 2007), often resulting in increased emigration and mortality, and reduced growth (Grant and Kramer 1990). As intraspecific competition increases, it may be expected that activity rates will increase in response to, for example, reduced food intake due to reduced availability and quality (Amundsen et al. 2007; Guénard et al. 2012), increased interference (Blanchet et al. 2008), reduced growth efficiency (Guénard et al. 2012), or the use of marginal habitats (Mobæk et al. 2012). Alternatively, it has also been suggested that in certain cases, animals may reduce activity to conserve energy if increased competition causes food to be limited or of poor quality (Borkowski 2000; but see Mobæk et al. 2012). To date, several studies on activity patterns of diverse taxa such as ruminants (Mobæk et al. 2012), land snails (Cameron and Carter 1979), fish (Marchand and Boisclair 1998; Vera et al. 2011), and insects (Bailey 1981; Schou et al. 2013) have yielded equivocal results on this topic. Other studies of activity patterns have increased intraspecific competition by varying resource abundance (Howerton and Mench 2014) or energetic requirements (Alanärä et al. 2001). However, observations conducted throughout the day/night cycle in relatively natural conditions are rare. Such an approach is necessary for revealing fine‐scale shifts in the timing of activity that may occur under conditions of increased competition and to understand how other ecological variables (e.g., water temperature) affect activity.

Stream salmonids are ideal for studying fine‐scale changes in diel activity because of their highly variable activity patterns and because they typically compete for food and space via territoriality and dominance hierarchies (Grant and Kramer 1990; Nakano 1995; Blanchet et al. 2008). Diel activity differs among species (Reebs 2002), populations (Valdimarsson et al. 2000), cohorts (Bradford and Higgins 2001), and individuals (Breau et al. 2007). By monitoring individual fish over an extended time, which is rarely done in the wild (but see Nakano 1995; Breau et al. 2007; Roy et al. 2013), activity and other behavior can be linked to individual growth rates, which ultimately affect individual survival and fitness (Smith and Griffith 1994). Many studies suggest competition as the primary explanation for the inverse relationship often reported between individual growth rates and density in juvenile salmonids (e.g., Jenkins et al. 1999; Imre et al. 2005), although such relationships are not always detected (e.g., Kaspersson et al. 2013). When competition increases through, for example, increased population density, temporal partitioning of resources may be a viable strategy for maintaining growth (Kronfeld‐Schor and Dayan 2003).

Juvenile (age 1+) Arctic charr *Salvelinus alpinus* were used to evaluate whether and how individuals modify the rate and timing of activity in response to increased population density and whether population density affects individual growth rates. Arctic charr exhibits flexibility in activity patterns and social behavior (Valdimarsson et al. 2000; Gunnarsson and Steingrímsson 2011), and has the northernmost distribution of any freshwater fish (Klemetsen et al. 2003). We tested the prediction that at high population density, fish increase their activity rate and spend more time foraging, for example, to counter increased interference and/or reduced food availability. Activity rates of age 1+ fish should be highest at night when predation risk is lower (sensu Imre and Boisclair 2004; Breau et al. 2007). However, because the cost of increased competition may outweigh the benefits of nocturnal foraging, fish at high density should distribute their activity over a greater portion of the 24‐h cycle than at low density. Concurrently, we explored whether and how activity is related to ecological conditions (e.g., fluctuations in water temperature, light intensity, and water depth). Finally, we expected that growth rates of fish at high density would be less than or similar to those of fish at low density, depending on the extent to which individuals increase their activity to compensate for increased competition (Blanchet et al. 2008; Guénard et al. 2012).

## Materials and Methods

### Experimental design

A field experiment was conducted in summer 2013 in a small side channel of Deildará, a runoff river in northern Iceland (65°50′54N, 19°12′55W). For more information on this system, see Gunnarsson and Steingrímsson (2011) and Tunney and Steingrímsson (2012). The experiment was repeated in time in two consecutive rounds, lasting from 3 to 17 July (15 days) and 20 July to 1 August (13 days). Four nylon mesh enclosures, suitable for behavioral observations (Lindeman et al. 2014), were planted in the stream in pairs, with approximately 70 m between the upstream and downstream pairs and 20 cm between adjacent enclosures. The enclosures (4 m long, 1 m wide, 0.75 m high) had a stretched mesh size of 5 mm, large enough not to significantly reduce the abundance of invertebrate drift (Keeley and Grant 1997; Zimmerman and Vondracek 2006), but small enough to prevent juvenile fish from escaping. String was stretched across the top of each enclosure to deter potential avian predators, presumably without affecting the risk perceived by fish. Within each enclosure, natural silt, sand, and gravel substrate (diameter <64 mm) were overlaid with cobbles (diameter = 64–250 mm) collected from the river bed. The substrate provided ample shelters for the study fish. To facilitate habitat mapping, a coordinate grid made from 1‐m metal poles (width = 8 mm) was placed on the streambed within each enclosure (Gunnarsson and Steingrímsson 2011). Bars were marked with tape at every 10 cm and positioned parallel and perpendicular to the enclosure length. Debris was removed from the sides of the enclosures as necessary.

Each pair of enclosures consisted of one enclosure stocked with 8 fish (2 fish/m^2^; low density) and another with 24 fish (6 fish/m^2^; high density). Pairing enclosures and alternating low‐ versus high‐density treatment enclosures between rounds ensured that other ecological variables were almost identical between treatments. The densities used were close to the average (1.50 fish/m^2^) and slightly above the maximum density (4.14 fish/m^2^) observed at a local scale for juvenile Arctic charr in three Icelandic streams (Gunnarsson and Steingrímsson 2011). At these densities, it is expected that competition should play a role in population regulation (Grant and Kramer 1990).

### Capture and tagging of study fish

A total of 128 wild 1+ Arctic charr (mean fork length ± SD: 60.0 ± 7.6 mm, range = 42.2–80.0; mean mass ± SD: 2.12 ± 0.93 g, range = 0.40–4.96) were electrofished in Deildará and its tributaries before the two experimental rounds (LR‐24 electrofisher; Smith‐Root, Inc., Vancouver, WA). Upon capture, fish were anaesthetized with phenoxyethanol and weighed to the nearest 0.01 g (PESOLA PPS200; CH‐6340 Baar, Switzerland). Fork length was measured with calipers to the nearest 0.1 mm. All eight fish in each low‐density enclosure and 9–10 fish in each high‐density enclosure were uniquely tagged with small subcutaneous injections of green, orange, red, or yellow visible implant elastomers (Northwest Marine Technology, Inc., Shaw Island, WA) in two positions along the dorsal fin (sensu Steingrímsson and Grant 2003). Tags spread vertically along the fin rays, were highly conspicuous, and remained visible throughout the experiment. Standardized mass‐specific growth rate (Ω; Ostrovsky 1995) was calculated for tagged individuals as Ω = ((*M*_Final_^*b*^–*M*_Initial_^*b*^)/(*b•t*))*•*100, where *M* is mass (grams); *b* is the allometric growth rate exponent, which adjusts for the scaling of metabolism with body size (see Sigourney et al. 2008); and *t* is the duration of the experimental round in days. The exponent *b* has not been estimated for Arctic charr, but has been estimated at 0.308 and 0.31 for brown trout (Elliott 1975) and Atlantic salmon (Elliott and Hurley 1997), respectively, suggesting that *b = *0.31 may be appropriate for salmonids generally (Quinn et al. 2004).

Fish were randomly distributed among the enclosures and allowed to habituate for 24 h before observations were made. A new group of fish was captured for the second round, following the procedure described above. After each round, fish in the enclosures were recaptured, measured for mass and length, and released in the area of initial collection. All fish except one untagged fish (high density) were recaptured alive, and all tagged fish were easily identified.

### Behavioral observations

Each enclosure was visited eight times per day (at 00:00, 03:00, etc.) during seven 24‐h cycles, yielding a total of 56 scans per enclosure during each round. Enclosures were visited in a random order at each time of day by one of three observers, who conducted 71%, 22%, and 7% of the scans, respectively. Any potential observer bias would have negligible effect on the main effect of density, because the effort of each observer was distributed equally between the two density treatments. Before scanning an enclosure, an observer stood motionless on the streambank for 10 min to ensure fish resumed normal behavior. The observer then recorded the number of fish and the identity of each tagged fish active within the enclosure. Each scan lasted <15 sec and was a “snapshot” of activity at a particular time. Fish were considered active if they searched for and/or attacked prey, either by holding a position against the current or actively swimming. Fish that were hiding in the substrate were considered inactive. Occasionally, fish rested completely motionless on the substrate (i.e., with no movement of the tail or pectoral fins), typically not facing the water current. These fish were never observed to forage from this position and were considered inactive at that time.

Bright summer nights in Iceland usually permit observations without the aid of artificial light. However, after a fish was located, a flashlight with a blue filter was occasionally used on cloudy nights to briefly enhance tags to ensure accurate identification. Artificial light rarely affected the focal fish, and any such disturbance occurred after a fish was determined to be active or not.

### Habitat measurements

Fluctuations in environmental conditions were monitored throughout the experiment (Table 1). Water temperature was recorded every hour by data loggers positioned at each pair of enclosures (Onset UTBI‐001 TidbiT v2; Onset Computer Corp., Bourne, MA). Light intensity was recorded hourly by a data logger positioned above the water surface at the upstream enclosure pair (Onset HOBO Pendant Temperature/Light 8K UA‐002‐08). Water level, an index of fluctuations in water depth, was measured to the nearest mm with a meter stick at a fixed location immediately upstream of each enclosure pair after each set of scans (i.e., at 3‐h intervals).

**Table 1:** Summary (mean and range) of habitat characteristics within each stream enclosure and between population density treatments during the 28‐day experiment conducted in Deildará, Iceland, in July 2013

Enclosure *or* treatment	Water temperature (°C)	Light intensity (kilolux)	Water depth (cm)	Current velocity (m/s)	Substrate size^a^	Food availability (items/m^3^)
1	5.2 (2.7–8.8)	35.21 (0.01–209.42)	18.3 (2.3–30.2)	0.09 (0.00–0.26)	5.6 (5–7)	13.9 (3.3–25.2)
2			17.5 (0.9–28.4)	0.10 (0.00–0.30)	5.6 (5–7)	10.6 (3.9–18.7)
3	5.5 (2.7–9.8)		15.8 (0.0–35.5)	0.10 (0.00–0.31)	5.7 (5–7)	17.8 (2.0–47.1)
4			17.8 (1.6–38.8)	0.10 (0.00–0.28)	5.7 (5–7)	13.8 (1.4–60.9)
Low	5.4 (2.7–9.8)	35.21 (0.01–209.42)	17.3 (0.0–35.5)	0.09 (0.00–0.31)	5.6 (5–7)	13.8 (1.4–47.1)
High			17.4 (0.0–38.8)	0.09 (0.00–0.28)	5.6 (5–7)	14.3 (2.0–60.9)

a Substrate size classified using a modified Wentworth scale (DeGraaf and Bain 1986): 1 – plant detritus; 2 – clay, < 0.004 mm; 3 – silt, 0.004–0.062 mm; 4 – sand, 0.062–2.0 mm; 5 – gravel, 2.0–64.0 mm; 6 – cobble, 64.0–250.0 mm; 7 – boulder, >250 mm; 8 – bedrock; 9 – macrophytes.

Water depth and current velocity were measured inside each enclosure near the beginning, middle, and end of each sampling round (Table 1). Water depth was measured at five points along 21 parallel transects perpendicular to the direction of stream flow (i.e., every 20 cm along both the x‐ and y‐axes of the coordinate grid). Current velocity at 40% water depth (from the substrate, sensu Davis and Barmuta 1989) was measured at four points along seven parallel transects perpendicular to stream flow with an electromagnetic flow meter (Flo‐Mate Model 2000; Marsh‐McBirney Inc., Frederick, MD). Dominant substrate particle size was quantified using a modified Wentworth scale (DeGraaf and Bain 1986) for sixty‐four 25 cm^2^ squares in each enclosure.

Food abundance was estimated by collecting invertebrate drift four times in each enclosure (00:00, 06:00, 12:00, 18:00) throughout four 24‐h cycles. Samples were taken 5–6 days into (8 and 24 July) and 3–4 days before the end of (14–15 and 30–31 July) each experimental round, for a total of 64 samples. A 250‐*μ*m drift net (net opening = 25 × 40 cm; net length = 100 cm) was placed in the downstream half of each enclosure for 10 min. Current velocity was measured in the center of the drift net mouth at 50% water depth. Samples were preserved in 70% ethanol and processed at Hólar University College. Food items in each sample were counted under a stereomicroscope and sorted into order and/or family. Because drift samples were composed primarily (mean = 93.8%) of small Chironomid larvae, as well as some Diptera pupae and adults, all sampled invertebrates were of edible size for the fish in this study (sensu Keeley and Grant 1997). Food availability was expressed as drift density, that is, the number of potential prey items per cubic meter of water (sensu Allan and Russek 1985).

### Statistical analysis

We used two approaches to analyze the data, based on two metrics of activity. First, we analyzed how tagged individuals differed in activity and growth between low‐ and high‐density treatments. Individual activity rates were calculated for tagged fish as the number of scans an individual was observed active divided by the total number of scans in the respective experimental round. Data on diel activity are cyclical by nature, so time values were transformed into angles for circular statistics (Batschelet 1981). Specifically, circular mean was used to determine the mean time of activity of each tagged fish, and circular standard deviation was used to assess how dispersed individual activity rates were over the 24‐h cycle. Mean time of activity could not be calculated for two tagged fish that were each observed active only at diametrically opposite times of day (e.g., 00:00 and 12:00), but these fish were included in the analysis of dispersion of activity. The effect of density on individual activity rates and dispersion of individual activity rates was tested using ANOVA. Because the experimental design was replicated both in space (two pairs of enclosures) and in time (two rounds), round and pair were included as random effects in the analyses to account for residual variation among rounds, pairs, and enclosures. Hence, ANOVAs were of the form: Response ~ Density + Error (Round + Pair). The potential effect of density on individual growth rate was evaluated using this model, but with individual activity rate, initial body mass, and an interaction term between density treatment and initial body mass included as covariates (i.e., ANCOVA). Circular ANOVA was used to compare the mean time of activity between treatments.

Second, to evaluate how activity was related to fluctuations in ecological conditions, we used a generalized linear mixed model including density treatment, water temperature, light intensity, water level, Julian date, and first‐order interactions (excluding density) as explanatory variables. Enclosure was included as a random factor. Values for each explanatory variable (except density treatment) were converted to standardized *z*‐scores. Overall activity rates, originally calculated as the number of active fish in a particular enclosure during a given scan divided by the total number of fish in the enclosure, were used in this analysis because they could be linked to ecological conditions at a particular time. Importantly, however, no fish were observed active during 49% of scans, which results in a distribution that violates assumptions for traditional modeling approaches. Thus, the model utilized a binomial distribution with a logit link function; that is, instead of using the original estimates of overall activity, activity was treated as a binomial variable and was rated as “0” when no fish was detected during a scan of a particular enclosure and as “1” when at least one fish was active. A similar approach is recommended for modeling abundance of rare species and other data with an inflated zero class (see Welsh et al. 1996). If activity is independent of density, the probability of detecting a single active fish should be three times higher at high density. To account for this, different activity thresholds were used for low (one active fish) and high density (three active fish) before activity was scored as “1” for a particular scan.

Akaike's information criterion corrected for small sample bias (AIC_c_) was used to evaluate candidate models (see Grueber et al. 2011 and references therein). Model uncertainty was accounted for through model averaging (Bartón 2014), including the top model (that with the lowest AIC_c_ score) and models within 2 ΔAIC_c_ values to calculate model‐averaged coefficients for each explanatory variable and to estimate relative variable importance (0.00–1.00) in relation to activity. All analyses were performed in R (version 3.3.1; R Core Team 2014) using packages “circular” (distribution of activity in time; Agostinelli and Lund 2013), “lme4” (generalized linear models; Bates et al. 2014), and “MuMIn” (model averaging; Bartón 2014).

## Results

**Figure 1:**
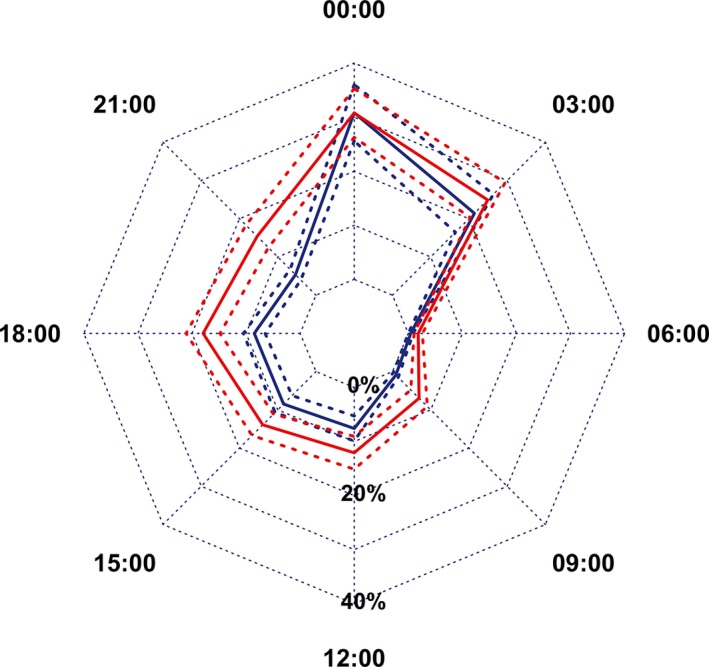
Diel distributions of individual activity rates of juvenile Arctic charr in Deildará, Iceland. Each *y*‐axis represents the mean individual activity rate (0–40%) for a given time (00:00, 03:00, etc.) of the 24‐h cycle. Blue and red lines represent low and high density, respectively. Dashed lines indicate standard error.

On average, individual tagged fish were active only 13.2% of the time (range = 0.0–55.4%), and 11 of the 71 tagged fish were never observed active. As predicted, individual activity rates were higher at high density (mean = 15.5%) than at low density (mean = 10.4%) (ANOVA, *F*_1,67_ = 4.475, *P *=* *0.038, Table 2). Fish in the second round (mean = 17.6%) were more active than fish in the first round (mean = 8.7%) (ANOVA, *F*_1,68_ = 12.65, *P *<* *0.001). In both density treatments, individual activity rates tended to be higher at night (i.e., from 21:00 to 03:00; high‐ and low‐density means = 22.3% and 16.5%) than during the day (i.e., from 06:00 to 18:00; high‐ and low‐density means = 8.7% and 4.4%; Fig. 1). There was no difference in the mean time of activity between treatments (circular ANOVA, χ^2^ = 0.686, *P *=* *0.407, Table 2), although fish in the second round were more nocturnal (mean time = 23:32) than fish in the first round (mean time = 19:37) (χ^2^ = 14.81, *P *<* *0.001).

**Table 2:** Patterns of activity and growth of individually tagged juvenile Arctic charr under treatments of high and low population density. Bold *P* values indicate significant differences between the two treatments

	Treatment						df	Mean Sq.	*F* value	*P* value
	High density			Low density						
	Mean	SD	*n*	Mean	SD	*n*				
Individual activity rate (%)^a^	15.5	13.9	39	10.4	9.3	32	1,67	0.045	4.475	**0.038**
Mean time of activity (h)^b^	21:59	3:32	32	22:49	3:49	26	1	*χ* ^2^ = 0.686		0.407
Dispersion of diel activity (h)^a^	4:39	2:36	34	3:03	2:23	26	1,56	0.003	4.639	**0.036**
Gs (% per day)^a^	1.6	0.9	39	1.5	0.9	32	1,64	<0.001	0.197	0.658

a Values obtained from ANOVA. These analyses included experimental round and enclosure pair as random effects, and the analysis on growth rate also included individual activity rate, initial body mass, and the interaction between density treatment and initial body mass as covariates. Refer to Appendix S1 for full ANOVA tables.

b Values obtained from circular ANOVA.

**Figure 2:**
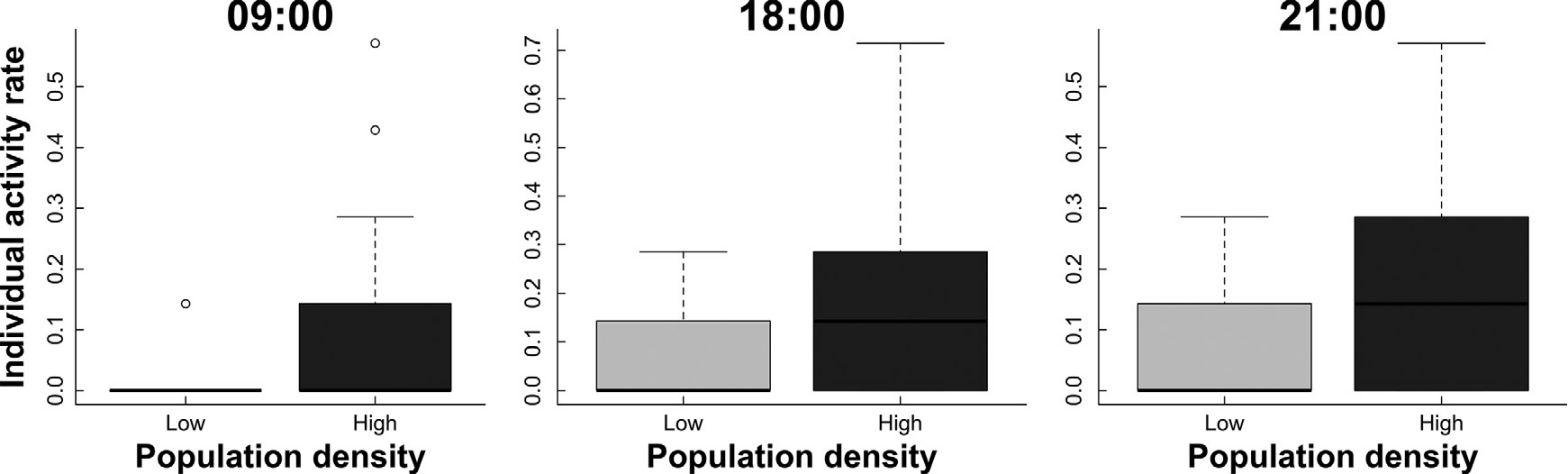
Individual activity rates at crepuscular times (09:00, 18:00, and 21:00) of juvenile Arctic charr in low and high population density treatments in Deildará, Iceland.

As predicted, individual activity was more dispersed over the 24‐h cycle at high density than at low density (ANOVA, *F*_1,56_ = 4.639, *P *=* *0.036; Table 2). Although the difference in individual activity rates between treatments was subtle, comparisons between treatments at each time period revealed two to eight times higher crepuscular activity at high density than at low density, specifically at 09:00 (ANOVA, *F*_1,67_ = 6.789, *P *=* *0.011), 18:00 (*F*_1,67_ = 6.800, *P *=* *0.011), and 21:00 (*F*_1,67_ = 8.788, *P *=* *0.004; Fig. 2).

**Figure 3:**
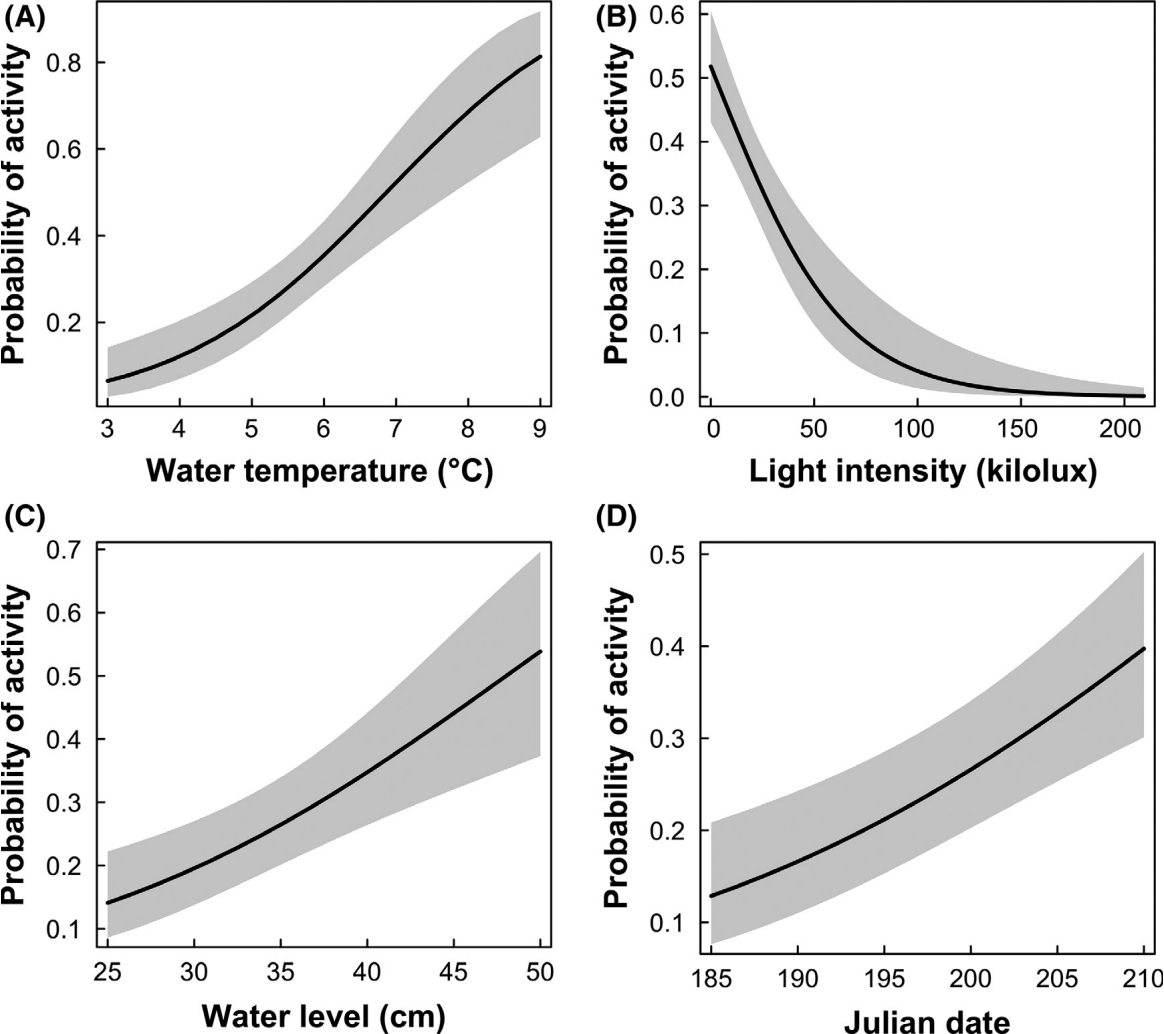
Effect size plots from model‐averaged generalized linear mixed model showing the probability of observing at least one or three fish (at low and high density, respectively) as a function of water temperature (A), light intensity (B), water level (C), and Julian date (D). Y‐axes have been rescaled to match the linear distribution of the independent variable. Shaded areas represent 95% confidence intervals.

**Figure 4:**
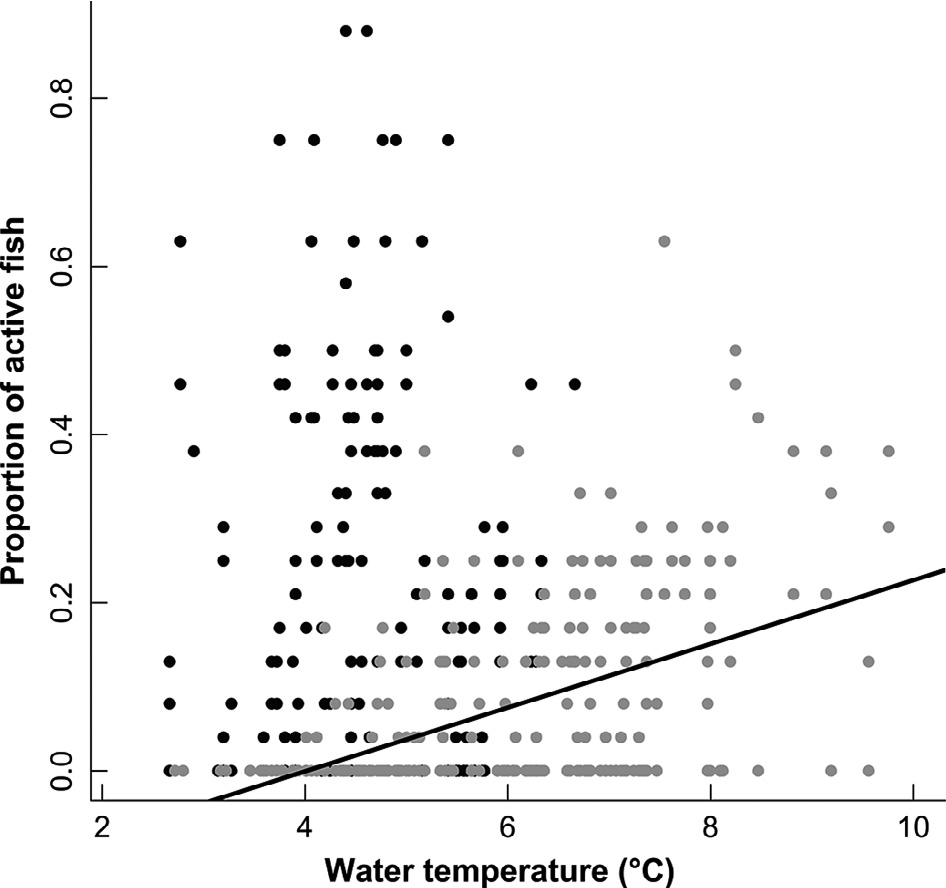
The association between activity levels of juvenile Arctic charr and water temperature (°C) in each stream enclosure during the day (i.e., 06:00–18:00; gray circles, solid line) and at night (i.e., 21:00–03:00; black circles).

By pairing low‐ and high‐density treatment enclosures in the stream, water temperature, light intensity, and water level were essentially identical between treatments. As well, water depth (ANOVA, *F*_1,3019_ = 0.548, *P *=* *0.459), current velocity (*F*_1,668_ = 1.336, *P *=* *0.248), and substrate size (*F*_1,508_ = 0.002, *P *=* *0.963) did not differ between treatments (Table 1). The model‐averaged generalized linear mixed model revealed that variability in activity within the study period was not only related to population density (*P *=* *0.003), but also to temporal fluctuations in other ecological variables. In fact, all single term variables included in the model had a significant impact on the probability of activity (Table 3). Fish were likelier to be active at higher population density, in warmer water, at higher water levels, and later in the season, but less likely to be active as light intensity increased (Fig. 3). The probability of activity was also affected by an interaction between water temperature and light intensity (Table 3). The proportion of active fish was positively correlated with water temperature during the day (Spearman's rank correlation, *n *=* *280, *P *<* *0.001), but not at night (*n *=* *168, *P *=* *0.477; Fig. 4).

**Table 3:** Results from model‐averaged generalized linear mixed model^a^ evaluating the effect of the population density treatment and ecological variables on the probability of detecting activity in juvenile Arctic charr, using a threshold of one and three fish at low and high density, respectively. Enclosure was included as a random factor. Bold *P* values indicate significant impact on activity

Source of variation	Relative importance	Estimate	SE	*Z* value	*P* value
Intercept	NA	−0.696	0.208	3.340	**0.001**
Treatment (low density)	1.00	−0.682	0.229	2.982	**0.003**
Water temperature	1.00	1.047	0.212	4.929	**<0.001**
Light intensity	1.00	−1.489	0.297	5.022	**<0.001**
Water level	1.00	0.457	0.122	3.741	**<0.001**
Julian date	1.00	0.494	0.130	3.786	**<0.001**
Water temp.*Light intensity	1.00	0.788	0.186	4.228	**<0.001**
Light intensity*Julian date	0.41	−0.087	0.143	0.607	0.544
Water temp.*Julian date	0.40	−0.077	0.133	0.584	0.560
Light intensity*Water level	0.25	0.027	0.072	0.373	0.709
Water temp.*Water level	0.23	0.026	0.076	0.343	0.731

a Model averaging based on ten candidate models all within 2 ΔAICc values. All ten models included each single term (i.e., treatment, water temperature, light intensity, water level, and Julian date) and the interaction between water temperature and light intensity, but variable combinations of other interactions. Refer to Table S1 for information on each candidate model and Figure S1 for a pairs plot of covariance between environmental variables.

There was no difference in food availability (i.e., invertebrate drift density) between treatments overall (ANOVA, *F*_1,60_ = 0.010, *P *=* *0.922). Drift density declined from the first round (mean = 17.4 items/m^3^) to the second round (mean = 10.4 items/m^3^) (Wilcoxon rank‐sum test, *n *=* *64, *P *<* *0.019). Drift density was significantly lower at 06:00 than at 12:00 (Wilcoxon rank‐sum test, *n *=* *16, *P *<* *0.001), and marginally lower than at 18:00 (*n *=* *16, *P *=* *0.068), but there were no differences in food availability between any other times of day (00:00, 12:00, 18:00; Fig. 5).

**Figure 5:**
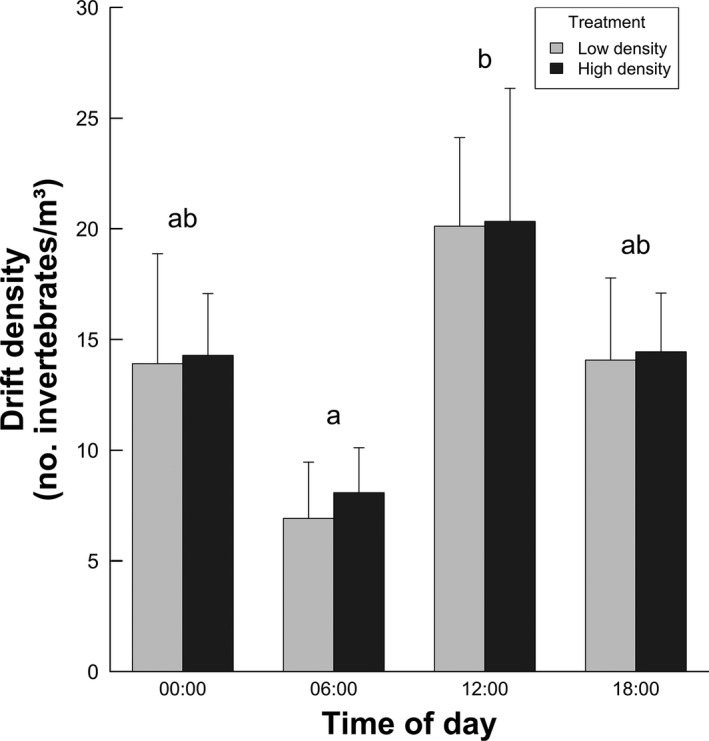
Density of invertebrate drift (mean ± SE) at different times of day under treatments of low and high population density of juvenile Arctic charr in Deildará, Iceland. Significant differences among times of day (Wilcoxon rank‐sum test, *P *<* *0.05) are identified with different letters.

Fish grew at a similar rate in low‐ and high‐density treatments (ANCOVA, *F*_1,64_ = 0.197, *P *=* *0.658; Table 2), with no difference in variance between treatments (Levene's test for homogeneity of variance, *F*_1,69_ = 1.083, *P *=* *0.302). Initial mass (ANCOVA, *F*_1,64_ = 3.118, *P *=* *0.082) and individual activity rate (*F*_1,64_ = 2.272, *P *=* *0.137) had no effect on growth rate (Appendix S1).

## Discussion

### Diel activity and population density

This study demonstrates that individuals can modify both the rate and temporal distribution of their activity in response to increased population density, with fish (1) increasing their activity rate and (2) extending their activity over a greater range of the 24‐h cycle. Recent studies on a variety of taxa show that population density may affect activity rates (e.g., domestic sheep *Ovis aries*, Mobæk et al. 2012; houseflies *Musca domestica*, Schou et al. 2013), but this has not commonly been examined in fish. In two separate studies, the proportion of juvenile Atlantic salmon *Salmo salar* (Armstrong and Griffiths 2001) and adult bullhead *Cottus gobio* (Davey et al. 2009) occupying shelters decreased with increased population density. This trend was explained by increased competition for limited shelters at higher densities, but indirectly suggests that activity may increase with population density. In the present study, activity rates increased with population density even though shelters were abundant.

Alternatively, Blanchet et al. (2008) found no significant intraspecific effect of population density on the activity of juvenile Atlantic salmon in stream channels, but these findings were based on short observations (5 min each) during narrow time intervals (9:00–11:00 and 20:30–22:30). Activity monitored on a regular basis throughout the 24‐h cycle under seminatural conditions should yield a more comprehensive test of density‐dependent activity patterns. In our study, dramatic differences in activity rates between density treatments were observed only at particular times of day. Although some studies on density‐dependent activity patterns have been conducted over 24‐h cycles (e.g., Cameron and Carter 1979; Bailey 1981; Bahrndorff et al. 2012), this has rarely been done in natural or seminatural conditions (but see Coulombe et al. 2008) or with fish (but see Vera et al. 2011 for an aquaculture study). Temporal shifts in activity patterns may occur when intraspecific competition increases due to temporal heterogeneity of resources (Craig and Douglas 1984), reduced resource availability (Hansen and Closs 2005; Howerton and Mench 2014), or in response to increased energetic requirements (Alanärä et al. 2001). The idea that intraspecific competition may induce shifts in the timing of activity has, in our opinion, not been addressed at sufficient temporal resolution throughout the day/night cycle.

In this study, Arctic charr were more active at night, with no difference in the mean time of activity between density treatments. However, at high density, fish distributed their activity over a greater portion of the 24‐h cycle, in part through increased activity at crepuscular times. This suggests that competition for drifting prey and/or interference from other fish may have prevented some individuals from being exclusively nocturnal, although aggression was observed in both low‐ and high‐density enclosures (A. Fingerle, pers. obs.). In a similar study of juvenile Arctic charr, competition for limited shelters resulted in increased and more dispersed activity (Larranaga and Steingrímsson 2015). Foraging at low light levels may benefit stream salmonids via reduced predation risk (Metcalfe et al. 1999) and lower rates of aggression (Fraser et al. 1993; Valdimarsson and Metcalfe 2001). In contrast, diurnal activity in this system may be risky due to increased vulnerability to predators (Webb 1978). Therefore, crepuscular times may represent a trade‐off between increased competition at night and higher predation risk during the day.

### Other ecological correlates of activity

The model‐averaged approach confirmed that fish are more active at high population density, but also showed that other ecological variables play key roles in shaping activity patterns. First, activity increased with water temperature, likely due to increased metabolic demands (Beamish 1964) as well as increased prey capture (Watz and Piccolo 2011) and position‐holding (Graham et al. 1996) abilities. In this study, metabolic demands were likely low because of low water temperatures, resulting in lower activity rates (mean = 13.2%) than have been observed in juvenile salmonids in warmer streams (e.g., mean = 36.8% in Breau et al. 2007; 23% in Roy et al. 2013). In all three studies, activity increased with rising temperatures, although activity may level off at extreme temperatures (e.g., 23°C for Atlantic salmon in Breau et al. 2007). Importantly, in spite of low temperatures and activity rates, the mean densities even of only active fish were still high enough to expect competition under natural conditions (Grant and Kramer 1990; Imre et al. 2005). For example, if we assume active fish occupied an average territory of 0.558 m^2^ (sensu Gunnarsson and Steingrímsson 2011), the mean habitat saturation at low (PHS = 11.6%) and high (PHS = 51.9%) density yields an 18.1% and 75.5% chance of density‐dependent mortality, growth, or emigration (sensu Grant and Kramer 1990). This suggests that activity should be taken into account when examining how animals share and compete for habitats, especially in colder regions where activity is generally low.

Second, activity decreased with light intensity. Although salmonids are visual foragers (see Rader et al. 2007) and have higher feeding efficiency (i.e., food intake vs. metabolic expenditure; sensu Metcalfe 1986) at daytime light levels (Fraser and Metcalfe 1997; Watz et al. 2014), fish in this study were more active at night. Salmonids tend to switch from diurnal feeding during their first year of life to more nocturnal feeding later in the juvenile phase. Our findings on 1+ fish, coupled with previous studies (Imre and Boisclair 2004; Breau et al. 2007), are thus consistent with the asset protection principle (Clark 1994), which states that animals with higher levels of reproductive assets, such as larger body size, should be less willing to risk predation.

Interestingly, water temperature and light intensity interacted in their effect on activity. More specifically, fish were more likely to be active during warmer rather than colder days, as has been found in previous studies (e.g., Gries et al. 1997; Breau et al. 2007; Blanchet et al. 2008), whereas activity levels at night were independent of water temperature (see also Fraser et al. 1993). The ability of fish to avoid predatory attacks decreases at colder temperatures, but so do gastric evacuation rates (Elliott 1972). Hence, fish may be able to preferentially hide from predators during colder days without sacrificing growth.

Third, activity also increased with rising water levels. Foraging in deeper water may increase prey encounter rate (Piccolo et al. 2007) and provide protection from aerial predators (Bugert and Bjornn 1991; Gregory 1993). However, high water levels may also coincide with fast current velocity and high turbidity, and thus, the benefits of foraging may be outweighed by the costs of swimming, causing fish to seek refuge. In a previous study, juvenile Arctic charr were found to be active at a mean water depth of 37.7 cm and at current velocities up to 39.9 cm/s (Tunney and Steingrímsson 2012). Thus, in the present study, water levels may have remained within the range of usable current velocities, resulting in a gradual increase in activity with increased water discharge. Fourth, fish became more active as the season progressed, even after accounting for any effect of water temperature, light intensity, and water level. One potential explanation may be food availability, which significantly decreased from the first (mean = 17.4 items/m^3^) to the second round (mean = 10.4 items/m^3^). Hence, fish may have spent more time foraging later in the season to capture enough prey to meet their energetic requirements.

### Population density, activity, and growth

Population density did not affect growth rates. Although many studies suggest density‐dependent growth in juvenile salmonids (e.g., Jenkins et al. 1999; Imre et al. 2005; Lindeman et al. 2014), this result is congruent with other studies that did not detect such an effect (e.g., Kaspersson et al. 2013). Two extremes along a continuum can be proposed for the way activity may shape the relationship between population density and individual growth. At one extreme, individuals at high density could compensate for increased interference (e.g., aggression and territorial defense; sensu Keddy 2001) or reduced food availability as a result of exploitative competition (e.g., shadow competition; Elliott 2002) by spending more time foraging. At the other extreme, individuals could show no flexibility in diel activity patterns and thus grow slower as a result of increased competition. In this study, fish at high density adjusted both the rate and timing of their activity and grew as fast as fish at low density. This suggests that in our study system, the former scenario is more likely, which reflects the findings of previous studies that suggest compensatory behavioral responses to changes in competition (Alanärä et al. 2001; Blanchet et al. 2008) and reduced food availability (Nicieza and Metcalfe 1997; Orpwood et al. 2006). Other compensatory mechanisms (i.e., changes in physiology that allow growth to be maintained, see Reznick et al. 2012) are also possible and deserve further exploration. To what degree increased activity levels allow individuals to maintain growth over a greater range of densities and over a longer period of time remains to be examined.

## Conclusions

This study suggests that intraspecific competition is important in shaping diel activity patterns of stream‐dwelling salmonids and animals in general. Using densities high enough to expect high levels of competition (Grant and Kramer 1990; Blanchet et al. 2008), we found that juvenile Arctic charr modified both the rate and timing of their activity and, consequently, maintained growth at higher population density. Ultimately, the difference in activity rates between high and low population density treatments was subtle although significant, and fluctuations in ecological conditions were also important in shaping activity patterns. Future research should attempt to tease apart the interplay between population density and ecological determinants in their effect on activity patterns, as the effect of density may ultimately depend on other ecological variables and could be intensified by, for example, high water temperature and low food availability. This study demonstrates that to obtain a more comprehensive understanding of the role of competition within populations, it is not only necessary to examine spatial patterns (e.g., territory size), but also how individuals modify temporal aspects of their foraging activity to gain access to resources and maintain growth.

## Conflict of Interest

None declared.

## Supporting information


**Figure S1.** Pairs plot of covariance between environmental variables included in the model‐averaged generalized linear mixed model, evaluating the effect of population density and ecological variables on the probability of detecting activity in juvenile Arctic charr.

**Table S1.** Candidate models included in the model‐averaged generalized linear mixed model, evaluating the effect of population density and ecological variables on the probability of detecting activity in juvenile Arctic charr.

**Appendix S1.** ANOVA tables testing for the effect of density treatment on individual activity, dispersion of activity, and individual growth rate.
