# Supplementary figures and images for: Density‐dependent diel activity in stream‐dwelling Arctic charr Salvelinus alpinus
Source: Ecol Evol. 2016 May 13;6(12):3965–76. doi: 10.1002/ece3.2177 (PMC4867681; doi:10.1002/ece3.2177)

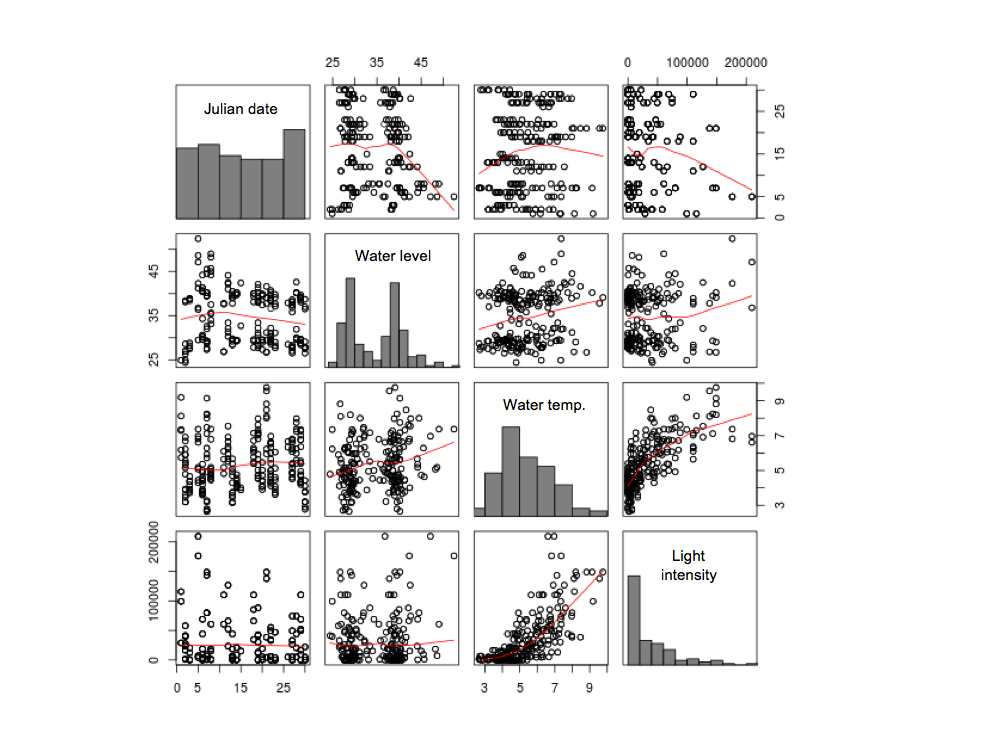

Supplement: Supplementary file 1 — Figure S1. Pairs plot of covariance between environmental variables included in the model‐averaged generalized linear mixed model, evaluating the effect of population density and ecological variables on the probability of detecting activity in juvenile Arctic charr. [file ECE3-6-3965-s001.tif]
